# Association of *MUTYH* and colorectal cancer

**DOI:** 10.1038/sj.bjc.6603239

**Published:** 2006-06-27

**Authors:** A Tenesa, H Campbell, R Barnetson, M Porteous, M Dunlop, S M Farrington

**Affiliations:** 1 Colon Cancer Genetics Group, University of Edinburgh, Western General Hospital, Crewe Road, Edinburgh, UK; 2 MRC Human Genetics Unit, Western General Hospital, Crewe Road, Edinburgh, UK; 3 Public Health Sciences, University of Edinburgh, Teviot Place, Edinburgh EH8 9AG, UK; 4 Clinical Genetics Department, University of Edinburgh, Edinburgh EH4 2XU, UK

**Keywords:** MUTYH, BER, MYH, colorectal cancer

## Abstract

Mutations in the *MUTYH* gene have been reported to be associated with increased risk of developing colorectal cancer. In this study, we confirmed this association using original data on 928 colorectal cancer cases and 845 healthy controls from Scotland. We then conducted a meta-analysis from published data on the association between mutations at *MUTYH* and colorectal cancer risk. We show for the first time a small but significant mono-allelic effect with a genotype relative risk (GRR) of 1.27 (95% confidence interval (CI): 1.01–1.61), and confirm and give a more precise estimate of the strong bi-allelic effect with an estimated GRR of 117 (95% CI: 74–184). This study underscores the need for large sample sizes in order to identify small gene effects when the disease allele frequency is low.

DNA repair genes are strong candidate cancer susceptibility genes (Ames and Gold, 1991; Yoshimura *et al*, 2003). One such gene is *MUTYH*, which together with *OGG1* and *MTH1* is a key component of the base-excision repair (BER) pathway. The main function of the BER pathway is to repair DNA oxidative damage caused by aerobic metabolism. Hence, *MUTYH* has raised much interest among those trying to unravel the genetic contribution to cancer risk (Al-Tassan *et al*, 2004; Tao *et al*, 2004; Farrington *et al*, 2005).

*MUTYH* has been associated with multiple colorectal adenomas (Al-Tassan *et al*, 2002) and with colorectal adenocarcinomas (Croitoru *et al*, 2004; Farrington *et al*, 2005). Here, we present a replication study in the Scottish population and a meta-analysis of published case–control data on *MUTYH* and colorectal cancer. Both types of study are important, because genetic association studies involving complex traits are frequently inconclusive owing to the difficulty of isolating the causal variant effect from other confounding factors. Hence, replication of the original findings is needed to support the validity of the association. Nevertheless, this might not always be possible, as unrealistically large follow-up studies would be required to detect small gene effects and in such instances the meta-analysis of published data may clarify the credibility of any inconclusive association.

## MATERIALS AND METHODS

### Replication data

We previously reported an association between *MUTYH* and colorectal cancer in a population-based study of 2239 cases (with histologically confirmed adenocarcinomas) and 1845 controls in Scotland. *MUTYH* homozygous mutation carriers had a 90-fold excess risk, whereas heterozygous carriers had no significant increased risk compared to wild-type homozygous on the overall cohort (Table 1), although there was a significant heterozygous effect in the late-onset cohort. Here, we extend this population-based study to include a further 928 colorectal cancer cases and 845 healthy controls. These samples were collected from the general population of Scotland. Cases were ascertained through all surgical Units in Scotland dealing with colorectal cancer. All cases had histologically confirmed adenocarcinoma of the colon or rectum. Blood DNA samples were obtained from the patients after fully informed consent. The study is subject to approvals from the Multi Centre Research Ethics Committee and all relevant Local Ethics Research Committee, as well as approval from NHS R&D Management for every participating hospital.

The two most common *MUTYH* variants in the Scottish population, Y165C and G382D, were genotyped following methods described previously (Farrington *et al*, 2005). Genotypes were coded as MM if the person was an Y165C/Y165C or G382D/G382D homozygote or Y165C/G382D or other compound heterozygote (detected by sequencing the entire coding region of the second allele of G382D/Y165C heterozygotes); as WM if the person was an Y165C or G382D heterozygote and otherwise as WW. All cases and 67% of controls were screened for a second mutation if they happened to be an Y165C or G382D heterozygote. Owing to the low frequency of compound heterozygotes other than Y165C/G382D in cases (1/12 in our original report) this is expected to have little effect on the analysis that follows.

### Meta-analysis data

In order to identify all relevant studies for the meta-analysis of the effect of *MUTYH* on colorectal cancer risk, we searched ISI Web of SCIENCE (http://wok.mimas.ac.uk/) and PUBMED (http://www.ncbi.nlm.nih.gov/entrez/query.fcgi) for the relevant literature references. We found 55 studies (searching for ‘MYH and colorectal cancer’), but only eight studies contained data that met our inclusion criteria and thus were relevant to this meta-analysis. These criteria were that patients had confirmed colorectal adenocarcinoma (i.e. we excluded studies only based on the multiple adenoma phenotype), and that the study reported genotype data for cases and controls (Table 2). Table 3 includes information on how the samples for each of the studies included in the meta-analysis were collected. Individuals reported to have two defects at *MUTYH* in the original report were classified as MM (defects were considered pathogenic only if there was published evidence of their pathogenicity), those with one defect as WM and those with no detected defect as WW. In total, we assembled data on 13 449 people: 7273 colorectal cancer patients and 6176 population-based controls.

### Statistical methods

The association of *MUTYH* with colorectal cancer was tested by means of a standard *χ*^2^ test with 2 degrees of freedom, and genotype relative risks (GRRs) were estimated as described previously (Farrington *et al*, 2005).

We used the metabin option from the meta package of the R software to perform the meta-analysis. We used the summary measure relative risk and the inverse variance weighting to pool studies. For mathematical reasons, cells with zero frequencies were assumed to be 0.5 (as defaulted by the meta package). For the recessive model with zero cell counts, we estimated exact confidence intervals (CIs) using Fisher's exact test.

## RESULTS

### Replication study

Table 1 shows the results obtained in this replication study, from our previously published data and from the combined data set. The replication study showed a significant homozygous MM effect (*P*<0.05), and the results were suggestive of an association with the *MUTYH* gene as a whole (*P*=0.09). Combining these data with our previously published data revealed a highly significant association with *MUTYH* (*P*=0.0006). However, a heterozygous effect was not detected (Peterlongo *et al*, 2005; Tenesa *et al*, 2005).

### Meta-analysis

First, we tested the overall association of the gene with colorectal cancer assuming three plausible genetic models: multiplicative, dominant and recessive. We found that the association was highly significant (*P*⩽0.0004) under the three genetic models considered. We did not find significant heterogeneity between studies under any of the models considered. The value of *I*^2^, which quantifies the level of heterogeneity on a continuous scale (Higgins and Thompson, 2002), was 0% (95% CI: 0.0–48.7), 0% (95% CI: 0.0–45.9) and 0% (95% CI: 0.0–39.8) for the multiplicative, dominant and recessive model, respectively. Therefore, we assumed that the effect was the same across studies and used a fixed effects model. Note, however, that using a random effects model gave identical results.

Next, we tested whether there was a homozygous (MM) and heterozygous (WM) gene effect (Table 4). The overall homozygous effect was highly significant (*P*=0.0004), whereas the overall heterozygous effect was almost significant (*P*=0.09).

To address the possibility that methodological differences across studies may have influenced these results, we pooled all available data described in Table 2 and estimated the GRRs as before (Farrington *et al*, 2005). We considered this was justified, as there was no statistically significant heterogeneity among studies. We performed the analyses both for all the 13 449 samples (data set 1) and separately for the 7657 samples not generated by our group (data set 2). The 95% CI for the GRR estimates in the two data sets overlapped both for MM and WM individuals. Data set 1 gave GRR for the MM and WM equal to 117 (95% CI: 74–184) and 1.27 (95% CI: 1.01–1.61), respectively. The overall *MUTYH* variant frequency was 0.34%. Similarly, data set 2 gave GRR for the MM equal to 207 (95% CI: 109–415) and 1.47 (95% CI: 1.05–2.12) for WM. Both data sets showed a highly significant (*P*<0.001) homozygous effect and a heterozygous effect of borderline statistical significance (*P*<0.05).

Finally, we tested whether there were differences in the effect of Y165C and G382D mutations (Table 5). Estimates of the mono-allelic effect were similar and not significantly different at the 5% level. However, the bi-allelic effect was much larger for Y165C carriers than for G382D carriers, although this difference was not statistically significant.

## DISCUSSION

This study confirms, in a combined data set of over 7000 cases across several study populations, that the association between variants in the *MUTYH* gene and colorectal cancer risk is valid. Bi-allelic inactivation of the gene conferred a very large increase in risk (GRR=117) supporting its causal role in colorectal cancer, whereas mono-allelic inativation of the gene conferred a moderate increase in risk (GRR=1.3). Confidence intervals for the estimate of the risk associated with germline bi-allelic defects were wide, even though the total sample size was over 13 000 individuals. This effect might be substantially overestimated if the proportion of compound heterozygotes in the control sample was equal to the case sample (i.e. 1/46), but we believe this is highly unlikely as we resequenced 32 (i.e. two-third) of our heterozygote controls and did not find any compound heterozygotes.

Similarly, mono-allelic defects are of borderline statistical significance. This underscores the important role of meta-analyses of large data sets from well-conducted studies to properly interpret these relationships. It is difficult to assess whether differences in the screening method employed by different studies might explain the small heterozygous effect (i.e. not all studies did a systematic screening of heterozygous individuals in order to discard other possible disease variants), but additional support from other studies (Jenkins *et al*, 2006) that used different methods and samples suggest that the small heterozygous effect is real. Hidden compound heterozygotes would further increase the numbers required to assess whether there is indeed a heterozygous effect because the heterozygous effect would be even smaller.

Our study confirms and provides more precise estimates for the homozygous effect and strengthens the evidence for a weak heterozygous effect. The study underscores some of the difficulties of studying the role of low-frequency variants of small effect and emphasises the need for international collaboration to achieve the very large sample sizes required to identify these variants and quantify their effects.

## Figures and Tables

**Table 1:** Numbers of cases and controls, GRR and empirical 95% CI from the population-based study in Scotland: data from the original report, the replication study and from the combined data^a^

	**Farrington *et al* (2005) (*P*=0.001)**		**Replication (*P*=0.09)**		**All together (*P*=0.0006)**	
**Genotype**	**No. of cases/controls**	**GRR (95% CI)**	**No. of cases/controls**	**GRR (95% CI)**	**No. of cases/controls**	**GRR (95% CI)**
MM	12/0	93.6 (42.6–213.2)	5/0	38.5 (10.6–110.1)	17/0	67.3 (35.3–128.7)
WM	45/28	1.4 (0.9–2.1)	18/20	0.8 (0.5–1.4)	63/48	1.13 (0.8–1.5)
WW	2160/1794	1.0	905/825	1.0	3065/2619	1.0
						
Total	2217/1822		928/845		3145/2667	

a GRR is the genotype relative risk, CI is the confidence interval and *P* is the significance level for the test of association using a *χ*^2^ test with 2 degrees of freedom.

**Table 2:** Association between *MUTYH* and colorectal cancer: review and meta-analysis of published data

**Study**	**No. of cases MM/WM/WW**	**No. of controls MM/WM/WW**	**Multiplicative model RR (95% CI)**	**Dominant model RR (95% CI)**	**Recessive model RR (95% CI)**
(Croitoru *et al*, 2004)	12/29/1197	0/21/1234	2.6 (1.6, 4.3)	2.0 (1.2, 3.4)	*∞* (2.84, ∞)
(Farrington *et al*, 2005)	12/45/2160	0/28/1794	2.0 (1.3, 3.2)	1.7 (1.1, 2.6)	*∞* (2.29, ∞)
(Peterlongo *et al*, 2005)	2/4/549	0/7/911	1.9 (0.7, 5.2)	1.4 (0.5, 4.2)	*∞* (0.31, ∞)
(Fleischmann *et al*, 2004)	2/5/351	0/0/97	4.1(0.2, 71.9)	4.2 (0.2, 72.2)	*∞* (0.05, ∞)
(Kambara *et al*, 2004)	0/2/90	0/1/52	1.2 (0.1, 12.6)	1.2 (0.1, 12.4)	NA
(Wang *et al*, 2004)	2/10/432	0/4/309	2.5 (0.8, 7.5)	2.1 (0.7, 6.6)	*∞* (0.13, ∞)
(Enholm *et al*, 2003)	4/5/994	0/0/424	11.5 (0.7, 193.1)	8.1 (0.5, 139.0)	*∞* (0.27, ∞)
(Zhou *et al*, 2005)	0/6/432	0/3/466	2.1 (0.5, 8.6)	2.2 (0.5, 8.6)	NA
Current replication study	5/18/905	0/20/825	1.3 (0.7, 2.3)	1.0 (0.6, 1.8)	*∞* (0.84, ∞)
Overall estimated effect	39/124/7110	0/84/6112	2.0 (1.6, 2.6)	1.6 (1.3, 2.1)	*∞* (8.63, ∞)

CI=confidence interval; NA=not available; RR=relative risk.

M *vs* W allele for the multiplicative model, MM and MW *vs* WW for the dominant model and MM *vs* MW and WW for the recessive model. The recessive model RR confidence intervals were estimated using Fisher's exact test.

**Table 3:** Information on the collection of samples of the studies included in the meta-analysis

**Study**	**Cases description**	**Population**	**Controls description**	**Population**
(Croitoru *et al*, 2004)	Prospective series	Regional Canadian	Age and sex matched	Same region of Canada
(Farrington *et al*, 2005) and replication	Prospective series	Scottish population	Age, sex and region matched	Scottish population
(Peterlongo *et al*, 2005)	Consecutive series	Regional USA	Chosen from 17 000 cohort study as cancer free and age matched	Same region USA and ethnically matched.
(Fleischmann *et al*, 2004)	Unrelated retrospective series	Three regions of UK	Spouses of individuals from another cancer study	No information
(Kambara *et al*, 2004)	Sporadic series	Regional Australia	Healthy blood donors	Same region of Australia
(Wang *et al*, 2004)	No information	Regional USA	Individuals negative by colonoscopy	Same region of USA
(Enholm *et al*, 2003)	Systematic series	Finnish population	Healthy blood donors	No information
(Zhou *et al*, 2005)	Sporadic series	Three regions of Sweden	Healthy blood donors	Stockholm region

**Table 4:** *MUTYH* genotype effect on colorectal cancer risk: review and meta-analysis of published data

	**Heterozygous (WM) effect**	**Homozygous (MM) effect^a^**
**Study**	**RR (95% CI)**	**RR (95% CI)**
(Croitoru *et al*, 2004)	1.4 (0.8, 2.5)	*∞* (2.86, *∞*)
(Farrington *et al*, 2005)	1.3 (0.8, 2.1)	*∞* (2.30, *∞*)
(Peterlongo *et al*, 2005)	0.9 (0.3, 3.2)	*∞* (0.31, *∞*)
(Fleischmann *et al*, 2004)	3.1 (0.2, 54.7)	*∞* (0.05, *∞*)
(Kambara *et al*, 2004)	1.2 (0.1, 12.4)	NA
(Wang *et al*, 2004)	1.8 (0.6, 5.6)	*∞* (0.14, *∞*)
(Enholm *et al*, 2003)	4.7 (0.3, 84.7)	*∞* (0.28, *∞*)
(Zhou *et al*, 2005)	2.2 (0.5, 8.6)	NA
Current replication study	0.8 (0.4, 1.6)	*∞* (0.83, *∞*)
Overall effect	1.3 (1.0, 1.7)	*∞* (8.63, *∞*)

CI=confidence interval; NA=not available; RR=relative risk.

a 95% CI estimated using exact methods (Fisher exact test).

**Table 5:** Counts of G382D and Y165C mutations reported in the published data and GRR

	**G382D**		**Y165C**	
**Study**	**Cases**	**Controls**	**Cases**	**Controls**
(Croitoru *et al*, 2004)	4/21/1197	0/17/1234	2/8/1197	0/4/1234
(Farrington *et al*, 2005)	8/31/2160	0/20/1794	0/14/2160	0/8/1794
(Peterlongo *et al*, 2005)	1/2/549	0/5/911	0/2/549	0/2/911
(Fleischmann *et al*, 2004)	1/4/351	0/0/97	0/1/351	0/0/97
(Kambara *et al*, 2004)	0/2/90	0/0/52	0/0/90	0/1/52
(Wang *et al*, 2004)	0/5/432	0/2/309	1/5/432	0/2/309
(Enholm *et al*, 2003)	1/4/994	0/0/424	0/1/994	0/0/424
(Zhou *et al*, 2005)	0/2/432	0/1/466	0/4/432	0/2/466
Current replication study	2/15/905	0/14/825	2/3/905	0/6/825
Overall numbers	17/86/7110	0/59/6112	5/38/7110	0/25/6112
GRR (MM/WM/WW)	103.63/1.26/1		168.82/1.31/1	
GRR 95% CI (MM/WM/WW)	(55.26–188.61)/(0.96–1.68)/(1.00–1.00)		(48.77–438.31)/(0.86–2.06)/(1.00–1.00)	

CI=confidence interval; GRR=genotype relative risk.
